# Bioactivities and Structure–Activity Relationships of Natural Tetrahydroanthraquinone Compounds: A Review

**DOI:** 10.3389/fphar.2020.00799

**Published:** 2020-05-27

**Authors:** Shixiu Feng, Weiyi Wang

**Affiliations:** ^1^Key Laboratory of South Subtropical Plant Diversity, Fairy Lake Botanical Garden, Shenzhen & Chinese Academy of Sciences, Shenzhen, China; ^2^Key Laboratory of Marine Biogenetic Resources, Third Institute of Oceanography, Ministry of Natural Resources, Xiamen, China

**Keywords:** tetrahydroanthraquinone, anti-cancer, structure–activity relationship, bioactivities, altersolanol A, bostrycin

## Abstract

Tetrahydroanthraquinones are a kind of important microbial secondary metabolites with promising biological activities. Most of them were found in microorganisms, a few were derived from Chinese herbal medicine. In this review, aiming to provide basis for the further research and development of tetrahydroanthraquinone compounds, we summarized the physiological activities of natural tetrahydroanthraquinone compounds, including anti-cancer, anti-microbial, and antidiabetic activities. The source, structure, and action mechanisms of active tetrahydroanthraquinones are described in detail. Furthermore, this review firstly analyzed the structure–activity relationship of tetrahydroanthraquinones. Our study will serve as a valuable guideline for further research on the structural optimization, mechanism study, and development of tetrahydroanthraquinone as novel drugs. Aiming to provide references for further studies and development of tetrahydroanthraquinone compounds.

## Introduction

Anthraquinones characterized by an anthraquinone scaffold structure, are widely distributed in plant as secondary metabolites. Modern pharmacological researcher showed that anthraquinones have various potent activities, including anticancer, anti-inflammatory, anti-injury, antibacterial, anti-osteoporosis, antioxidant, etc. (Li and Jiang, 2018).

**Figure 1:**
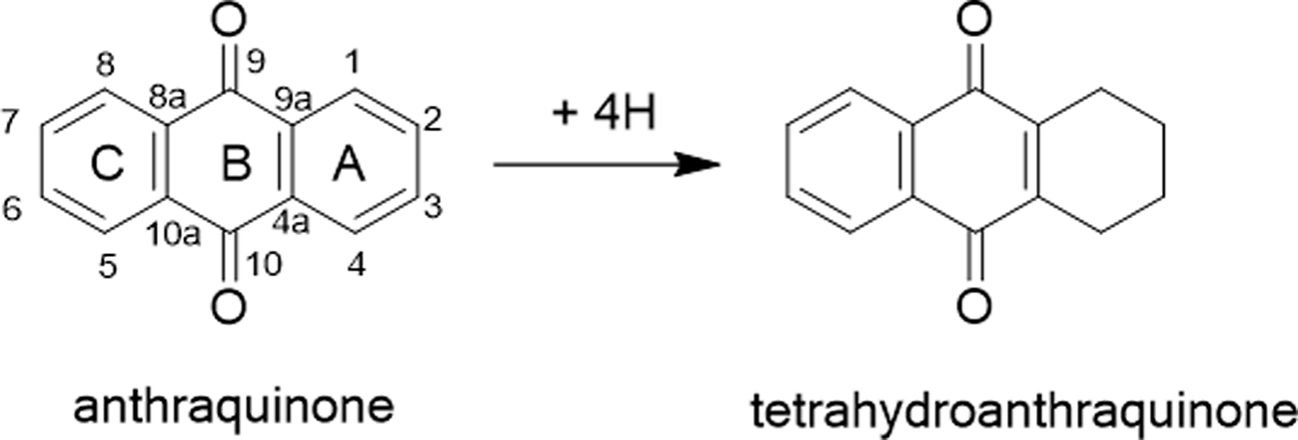
The structure of tetrahydroanthraquinone.

Tetrahydroanthraquinone is a class of derivatives of anthraquinone in which the unsaturated double-bonds on the benzene ring A are hydrogenated by four hydrogens (**Figure 1**). Most of them were found in microorganisms, while a little were derived from Chinese herbal medicine. To date, about 60 different tetrahydroanthraquinones are found, of which only nine are from plants (Wang C. et al., 2015). Because of some of tetrahydroanthraquinones showing good pharmacological activity, such as anticancer, antibacterial, anti-malarial, and anti-viral, more and more researchers are being focused on the exploration of their pharmacological activity, mechanism, and structure activity relationships.

Structurally, tetrahydroanthraquinones are generally classified into tetrahydro-9,10-anthraquinones, hydroxyphenanthrenes, tetrahydro-1,4-anthraquinones, and bi-tetrahydroanthraquinone, and according to their sources, tetrahydroanthraquinones can be divided into the ones from microorganisms and the ones from plants. In this paper, we focus on the pharmacological activities and related structure–activity relationships of active tetrahydroanthraquinones, aiming to provide references for further studies and development of tetrahydroanthraquinone compounds.

## Structure and Classification

### Tetrahydroanthraquinones From Microorganisms

#### Tetrahydro-9,10-Anthraquinones

**Figure 2:**
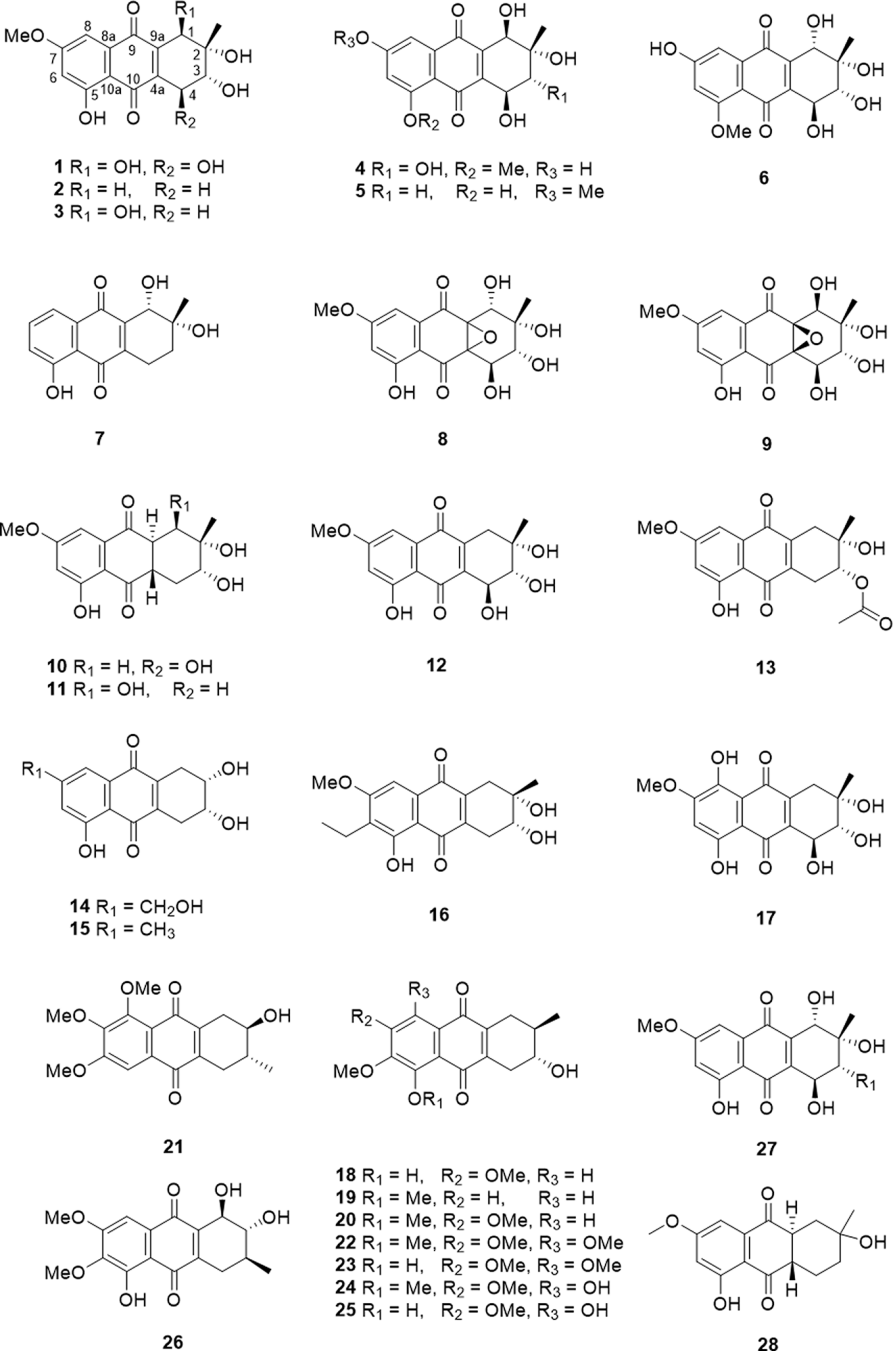
The structure of tetrahydro-9,10-anthraquinones compounds (1–28).

The tetrahydroanthraquinone ring is the fundamental parent nucleus of tetrahydroanthraquinones (**Figure 1**). Hydroxyls could locate in the C-1 to C-8 positions. In general, a compound with single tetrahydroanthraquinone nucleus is called a tetrahydro-9,10-anthraquinone. There are nineteen tetrahydro-9,10-anthraquinones that were isolated from endophytes and marine strains (**Table 1** and **Figure 2**). Besides, nine kinds of tetrahydro-9,10-anthraquinones extracted from the root of *Prismatomeris connata*, and they are named as prisconnatanones A-I (**Table 1** and **Figure 2**). Most tetrahydro-9,10-anthraquinones isolated from microorganisms have a structure with 5-OH and 7-OCH3, and substituent groups at A ring can be variable. However, tetrahydro-9,10-anthraquinones extracted from *P. connata* have a basic structure with 2-OH (R) and 3-CH3 (R), and their C ring mostly substituted with hydroxyl and methoxy groups.

**Table 1:** The source and activities of tetrahydro-9,10-anthraquinones compounds.

No	Name	Source	Activities	Reference
**1**	Altersolanol A	*Alternaria solani*	Antitumor activity; Antibacterial activity	(Stoessl, 1969a; Mishra et al., 2015; Zhang N. et al., 2016)
**2**	Altersolanol B	*A. solani*	Antibacterial activity	(Stoessl, 1969a; Yagi et al., 1993)
**3**	Altersolanol C	*Dactylaria lutea*	Antibacterial activity	(Becker et al., 1978; Yagi et al., 1993)
**4**	Altersolanol D	*A. solani*	–	(Yagi et al., 1993)
**5**	Altersolanol E	*A. solani*	Antibacterial activity	(Becker et al., 1978; Yagi et al., 1993)
**6**	Altersolanol F	*A. solani*	Antitumor activity	(Yagi et al., 1993; Bin et al., 2016)
**7**	Altersolanol P	*Staphylococcus aureus*	–	(Becker et al., 1978)
**8**	Altersolanol O	*Alternaria* sp. *XZSBG-1*	–	(Chen et al., 2014)
**9**	Auxarthrol C	*Stemphylium* sp. *33231*.	–	(Zhou et al., 2014)
**10**	Dihydroaltersolanol B	*Stemphylium globuliferum*	–	(Liu et al., 2015)
**11**	Dihydroaltersolanol C	*Stemphylium globuliferum*	–	(Liu et al., 2015)
**12**	4-dehydroxyaltersolanol A	*Nigrospora oryzae*	Antitumor activity; Hypoglycemic	(Uzor et al., 2015)
**13**	2-*O*-aectylaltersolanol B	*Stemphylium* sp. *33231*.	–	(Zhou et al., 2014)
**14**	Trichodermaquinone	*Trichoderma aureoviride PSU-F95*	Antibacterial activity	(Khamthong et al., 2012)
**15**	Coniothranthraquinone 1	*T. aureoviride PSU-F95*	Antibacterial activity	(Khamthong et al., 2012; Ng et al., 2015)
**16**	Phomopsanthraquinone	*Phomopsis* sp. *PSU-MA214*.	–	(Klaiklay et al., 2012)
**17**	SZ-685C	*fungus No. 1403*	Antitumor activity	(Xie et al., 2010; Zhu et al., 2012; Chen et al., 2013; Wang et al., 2013; Wang X. et al., 2015)
**18**	Prisconnatanone A	*Prismatomeris connata*	Antitumor activity	(Feng et al., 2011; Feng et al., 2016; Feng et al., 2018)
**19**	Prisconnatanone B	*P. connata*	–	(Feng et al., 2011)
**20**	Prisconnatanone C	*P. connata*	–	(Wang C. et al., 2015)
**21**	Prisconnatanone D	*P. connata*	–	(Wang C. et al., 2015)
**22**	Prisconnatanone E	*P. connata*	–	(Wang C. et al., 2015)
**23**	Prisconnatanone F	*P. connata*	–	(Wang C. et al., 2015)
**24**	Prisconnatanone G	*P. connata*	–	(Wang C. et al., 2015)
**25**	Prisconnatanone H	*P. connata*	–	(Wang C. et al., 2015)
**26**	Prisconnatanone I	*P. connata*	Antitumor activity	(Wang C. et al., 2015)
**27**	1,2,4,5-tetrahydroxy-7-methoxy-2-methyl- 1,2,3,4-tetrahydroanthracene-9,10-dione	*Alternaria* sp.	–	(Phunlap et al., 2013)
**28**	(±)-4-deoxyaustrocortilutein (4-DACL)	Derivative	–	(Genov et al., 2016)

#### Hydroxyphenanthrenes

**Figure 3:**
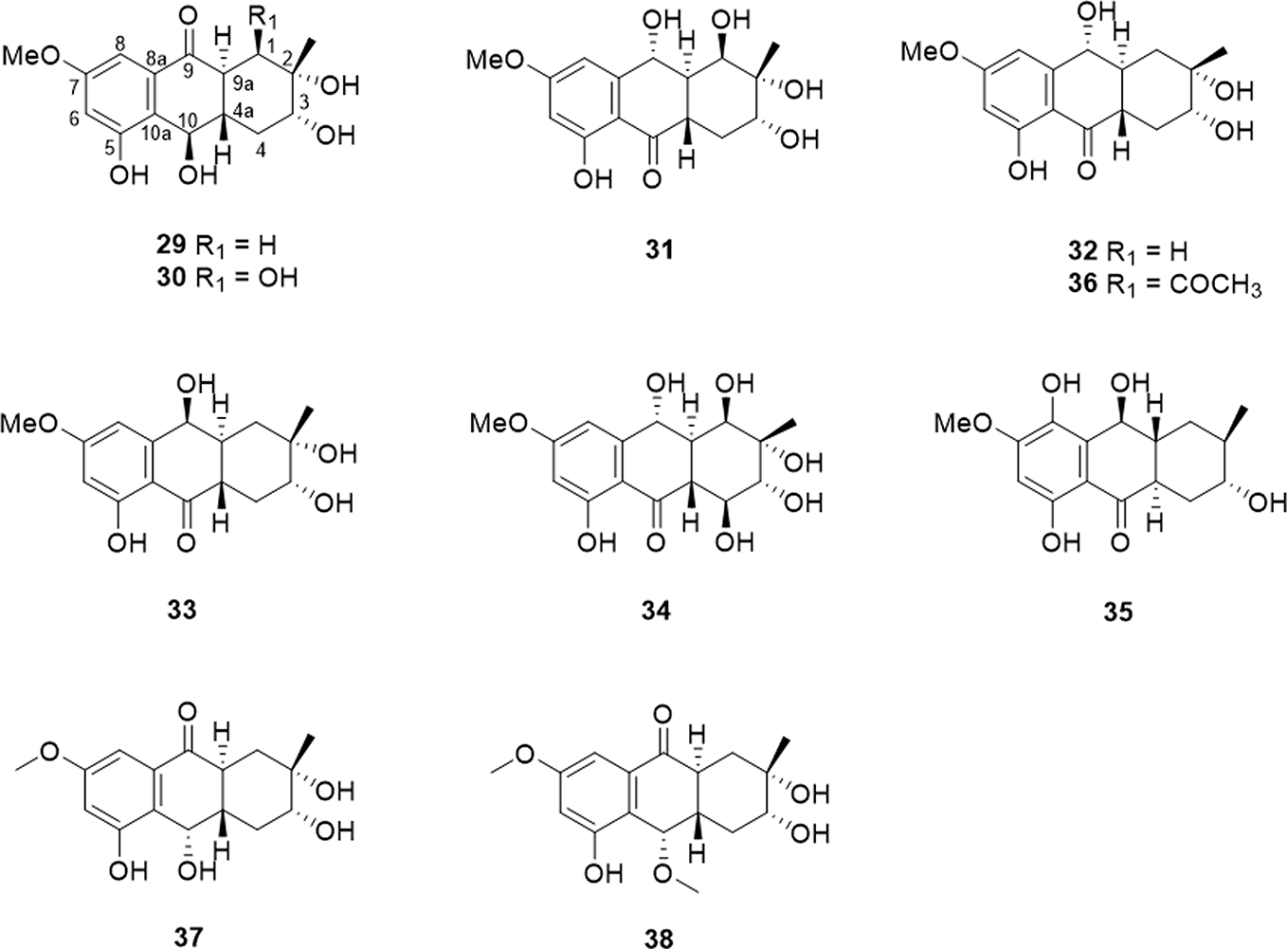
The structure of hydroxyphenanthrene compounds (29–38).

Hydroxyphenanthrene is a class of compounds in which the C-9 or C-10 position is hydrogenated to a hydroxyl group compared to a tetrahydroanthraquinone, and generally named as 10-hydroxy-1,3,4,4a,9a,10-hexahydrohydroanthracene-9(2H)-one. There are ten hydroxyphenanthrenes that has been isolated and identified. Their detailed source and structure information were displayed in **Table 2** and **Figure 3**.

**Table 2:** The source and activities of hydroxyphenanthrene compounds.

No	Name	Source	Activities	Reference
29	Altersolanol J	*NRRL 29097*	–	(Höller et al., 2002)
30	Altersolanol K	*Stemphylium globuliferum*	–	(Debbab et al., 2009)
31	Altersolanol L	*S. globuliferum*	–	(Debbab et al., 2009)
32	Tetrahydroaltersolanol B	*Phomopsis* sp. *PSU-MA214*	–	(Klaiklay et al., 2012)
33	Tetrahydroaltersolanol C	*Phomopsis* sp. *PSU-MA214*	Antiviral activity	(Klaiklay et al., 2012; Zhang S.L. et al., 2016)
34	Ampelanol	*Ampelomyces* sp.	–	(Aly et al., 2008)
35	Xylanthraquinone	*Xylaria* sp. *2508*	–	(Huang et al., 2014)
36	2-*O*-aectylaltersolanol L	*Stemphylium* sp. *33231*	–	(Zhou et al., 2014)
37	Altersolanol Q	*Stemphylium globuliferum*	–	(Moussa et al., 2016)
38	10-methylaltersolanol Q	*S. globuliferum*	–	(Moussa et al., 2016)

#### Tetrahydro-5,8-Anthraquinones

**Figure 4:**
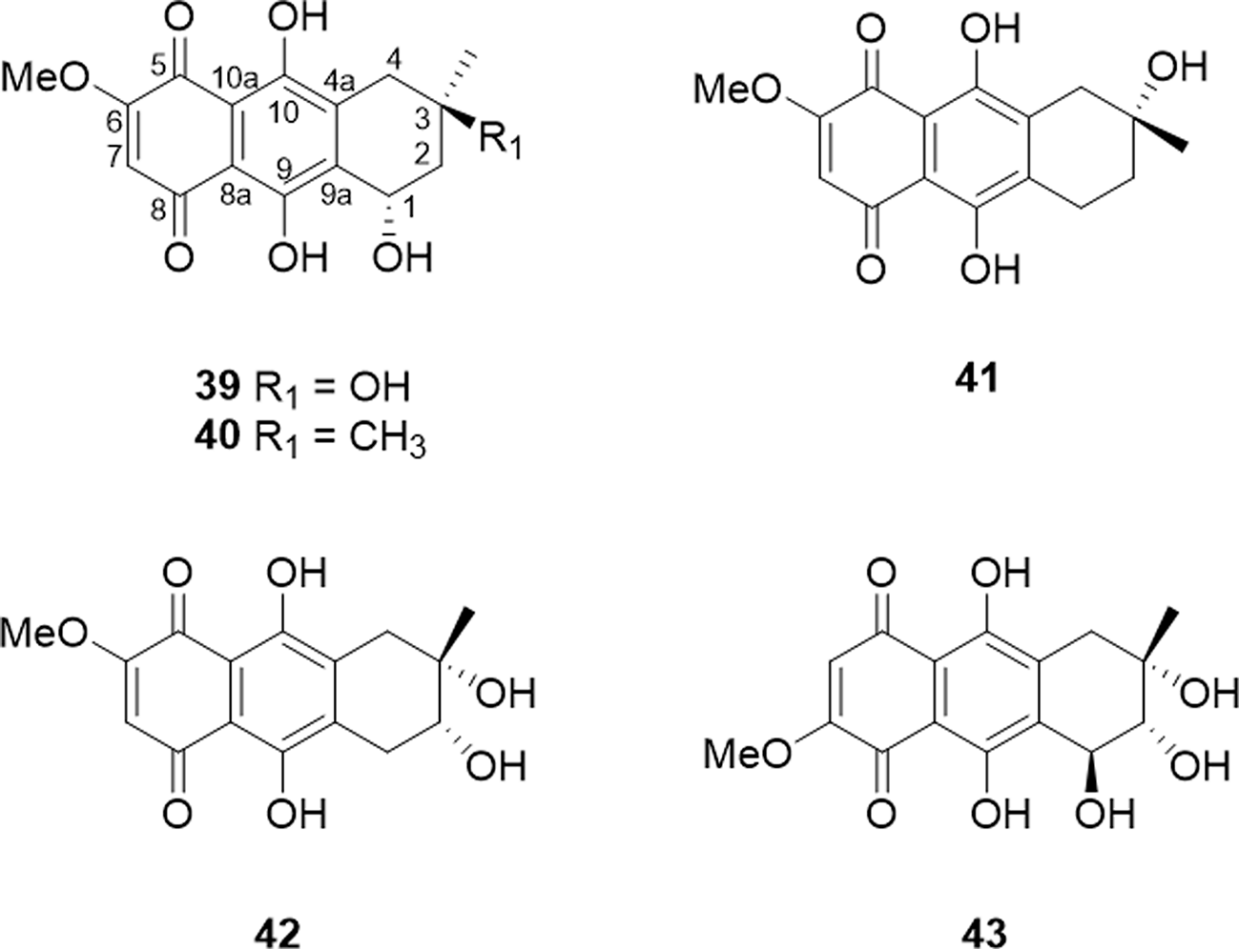
The structure of tetrahydro-5,8-anthraquinones compounds (39–43).

Tetrahydro-5,8-anthraquinones is a class of compounds in which the C5 and C8 positions of a tetrahydroanthraquinone are oxidized to a ketone group. They are rare in natural products due to their instability. In nature, most of anthraquinones and tetrahydroanthraquinones exist in the form of C9 and C10 ketone groups. Although rare, five Tetrahydro-5,8-anthraquinones have been isolated and identified as (1S,3R)-austrocortirubin (6-methoxy-1β,3β,9,10-tetrahydroxy-3α-methylanthracene-9,10-dione), (1S,3S)- austrocortirubin (6-methoxy-1β,9,10-trihydroxy-3α,3β-dimethylanthracene-9,10-dione), 1-deoxyaustrocortirubin (7-methoxy-2β,9,10-trihydroxy-2α-methylanthracene-9,10-dione), deoxybostrycin (7-methoxy-2β,3β,9,10-tetrahydroxy-2α-methylanthracene-9,10-dione), and bostrycin (7-methoxy-1α,2β,3β,9,10-pentahydroxy-3α-methylanthracene-9,10-dione). The detailed source and structure information was displayed in **Table 3** and **Figure 4**.

**Table 3:** The source and activities of tetrahydro-5,8-anthraquinones compounds.

No	Name	Source	Activities	Reference
39	(1*S*,3*R*)-austrocortirubin	*Dermocybe splendida*	–	(Elsworth et al., 1999)
40	(1*S*,3*S*)-austrocortirubin	*Cortinarius* sp	Antitumor activity	(Choomuenwai et al., 2012; Wang Y. et al., 2015)
41	1-deoxyaustrocortirubin	*Cortinarius* sp	–	(Choomuenwai et al., 2012)
42	Deoxybostrycin	*Xylaria* sp. 2508	Antitumor activity; Antibacterial activity; Antimalarial activity	(Xuekui et al., 2011; Cong et al., 2013; Huang et al., 2014)
43	Bostrycin	*Xylaria* sp. 2508	Antitumor activity; Antibacterial activity	(Dongni et al., 2013; Huang et al., 2014)

#### Bi-Tetrahydroanthraquinones

**Figure 5:**
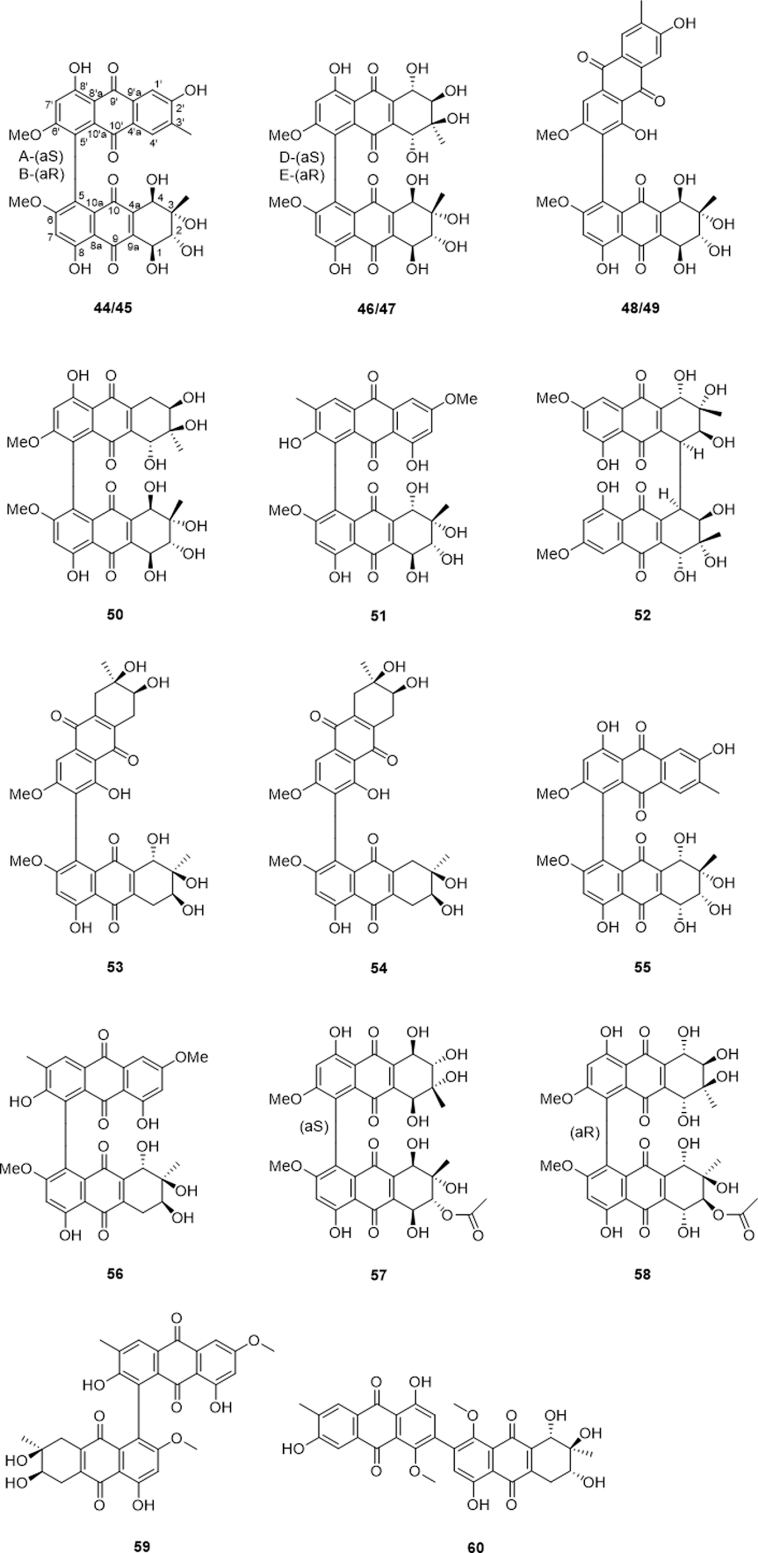
The name of bi-tetrahydroanthraquinones compounds (44–60).

Bi-tetrahydroanthraquinones were formed by two tetrahydroanthraquinones or a tetrahydroanthraquinone and an anthraquinone through dehydration condensation to establish dimeric alterporriols. Seventeen bi-tetrahydroanthraquinones have been reported (**Table 4** and **Figure 5**), among which based on the type of the monomeric units, biaryl linkage, presence of axial chirality, they could be further classified as anthraquinone connected to tetrahydroanthraquinone unit *via* C-5−C-5′ biaryl linkage (44, 45, 51, 55, and 56), and C-7−C-5′ biaryl linkage (48, 49, 59, and 60). As to the bi-tetrahydroanthraquinone units, compounds 46, 47, 50, 57, and 58 coupled by C-5−C-5′ biaryl linkage, compounds 53 and 54 presented a C-7−C-5′ biaryl linkage, only compound 52 featured a C-4−C-4′ cyclohexene connection. Moreover, compounds 50, 52, 53, and 54 demonstrated as axial chirality monomers, compounds 44 and 45, compounds 46 and 47, compounds 48 and 49 were atropodia steremoers.

**Table 4:** The source and activities of Bi-tetrahydroanthraquinones compounds.

No	Name	Source	Activities	Reference
44	Alterporriol A	*Stemphylium* sp. 33231	–	(Zhou et al., 2014)
45	Alterporriol B	*Stemphylium* sp. 33231	–	(Zhou et al., 2014)
46	Alterporriol D	*Pleospora herbarum*	–	(Kanamaru et al., 2012)
47	Alterporriol E	*P. herbarum*	–	(Kanamaru et al., 2012)
48	Alterporriol G	*Stemphylium globuliferum*	Antibacterial activity	(Aly et al., 2008; Debbab et al., 2009)
49	Alterporriol H	*S. globuliferum*	–	(Debbab et al., 2009)
50	Alterporriol F	*Pleospora herbarum*	–	(Kanamaru et al., 2012)
51	Alterporriol N	*Alternaria* sp. *XZSBG-1*	–	(Chen et al., 2014)
52	Alterporriol S	*Alternaria* sp. *XZSBG-1*	–	(Chen et al., 2014)
53	Alterporriol T	*Stemphylium* sp. 33231	–	(Zhou et al., 2014)
54	Alterporriol U	*Stemphylium* sp. 33231	–	(Zhou et al., 2014)
55	Alterporriol V	*Alternaria* sp. *XZSBG-1*	–	(Chen et al., 2014)
56	Alterporriol W	*Stemphylium* sp. 33231	–	(Zhou et al., 2014)
57	Aectylalterporriol D	*S. globuliferum*	–	(Liu et al., 2015)
58	Aectylalterporriol E	*S. globuliferum*	–	(Liu et al., 2015)
**59**	Alterporriol X	*S. globuliferum*	–	(Moussa et al., 2016)
**60**	Alterporriol L	*Alternaria* sp. *ZJ9-6B*	Antitumor activity	(Huang et al., 2012)

## Pharmacological Effects

### Anti-Tumor Activity

#### Anti-Tumor Activity of Tetrahydro-9,10-Anthraquinones

Some natural anthraquinones have been proven to have anti-tumor effects, such as rhein and emodin (Li and Jiang, 2018; Han et al., 2018). Tetrahydroanthraquinones, a class of anthraquinones, can also exhibit anti-cancer activity such as inhibiting cell proliferation, invasion, metastasis, and angiogenesis by inducing cell apoptosis, arresting cell cycle or suppressing the relevant enzymes. Altersolanol A, one of the most researched tetrahydroanthraquinone, exhibits antitumor activities against broad spectrum cancers (bladder, colon, gastric cancers, etc.) (Zhang N. et al., 2016). It can inhibit the proliferation and migration of both adherent cells K562 and non-adherent cells A549, whereas leave non-cancer cells (PBMCs cells) unaffected (Teiten et al., 2013). The anti-tumor activity of Altersolanol A is correlated with its pro-apoptotic and anti-invasive effect due to the inhibition of NF-κB transcription (Teiten et al., 2013). Another study also showed Altersolanol A exerted anti-cancer activity by inhibiting angiogenesis at low concentration *in vitro* and *in vivo*, and suppress the proliferation, tube formation, and migration of endothelial cells (Phunlap et al., 2013). Altersolanol F impaired the viability of colorectal cancer HCT-116 cells and cervical cancer Hela cells (Bin et al., 2016). 4-Dehydroxyaltersolanol A showed cytotoxicity against L5178Y mouse lymphoma cells with IC_50_ value of 9.4 μM (Uzor et al., 2015). Altersolanol N and alterporriol G were also cytotoxic towards the murine cancer cell line L5178Y (Aly et al., 2008). SZ-685C, isolated from *Halorosellinia* sp. (No. 1403), exhibits broad spectrum of antitumor activity. A study showed it inhibits the growth of human glioma, hepatoma, prostate cancer, and breast cancer cells with IC_50_ values ranging from 3.0 to 9.6 μM (Xie et al., 2010). *In vivo* experiment indicated that SZ-685C could suppress the tumor growth in nude mice by inducing apoptosis through the Akt/FOXO pathway (Xie et al., 2010). In addition, SZ-685C was reported to induce apoptosis in primary human nonfunctioning pituitary adenoma cells and adriamycin-resistant human breast cancer cells by inhibition of the Akt Pathway (Zhu et al., 2012; Wang X. et al., 2015). Moreover, recent studies found that it may play antitumor function through regulating the expression of micro RNAs. Chen et al. (2013) reported that SZ-685C inhibited the proliferation of rat pituitary adenoma MMQ cells and induced cell apoptosis through downregulating the expression of miR-200c. Dujuan Wang et al. (2013) suggested that SZ-685C abrogated the radio resistance of human nasopharyngeal carcinoma CNE2 cells through the miR-205−PTEN-Akt pathway. (±)-4-deoxyaustrocortilutein treatment induced mitochondrial ROS, reduced NF-κB signaling activity and increased up-regulation of the cell cycle inhibitors cyclin-dependent kinase inhibitor p21 (p21WAF1/Cip1) and the tumor suppressor protein p53 in a dose-dependent manner (Genov et al., 2016).

Prisconnatanones A and I were isolated from *Prismatomeris connata*, showed strong anticancer activity. Prisconnatanones A (HG30) inhibits the proliferation of HEp-2, A549, and H1299 cells, inducing apoptosis through caspase-dependent apoptosis pathways and disturbing the balance between Bcl-2 and IAP families, in addition, its cytotoxicity was associated with the cell cycle arrest at G2/M phase (Feng et al., 2016; Feng et al., 2018). Prisconnatanone I showed the highest activity, with IC_50_ values ranging from 2.7 µM to 3.9 µM in the suppression of lung tumor cell growth (H1229, HTB179, A549, and H520), while compounds Prisconnatanone C-H had relatively low values (IC_50_, 2.7 µM to 3.9 µM) (Wang C. et al., 2015). In conclusion, these data suggest that some natural tetrahydroanthraquinones are bioactive, and hydroxylation at C-1 significantly increases the cytotoxicity of these compounds against lung tumor cells growth.

#### Anti-Tumor Activity of Tetrahydro-5, 8-Anthraquinones

(1S,3S)-austrocortirubin, a tetrahydro-5, 8-anthraquinones, has a GI_50_ of 3 µM against colon cancer HCT116 cells and induces apoptosis *via* inducing DNA damage. It causes significant DNA damage during G0/G1, S, and G2 cell cycle phases. Cells are stopped at the G2/M phase checkpoint, and do not reach mitosis due to large amounts of DNA damage (Wang Y. et al., 2015). Deoxybostrycin exerted cytotoxicity against A549, Hep-2, Hep G2, KB, MCF-7, and MCF-7/Adr cells with IC_50_ values of 2.44, 3.15, 4.41, 3.15, 4.76, and 5.46 µg/ml, respectively. Bostrycin also inhibited the growth of these same cancer cell lines with IC_50_ values of 2.64, 5.39, 5.90, 4.19, 6.13, and 6.68 µg/ml, respectively (Xuekui et al., 2011). For bostrycin, previous study reported it inhibited cell proliferation *via* upregulation of miRNA-638 and miRNA-923 and downregulation of the PI3K/Akt pathway (Wei-Sheng et al., 2011). Besides, it could induce apoptosis and cell cycle arrest in A549 cells (Wei-Sheng et al., 2011). Further study uncovered that bostrycin inhibits the proliferation of breast cancer cells through changing the structure of PTP1B (protein tyrosine phosphatase 1B) and inhibiting its activity (Dongni et al., 2013).

To further explore the structure–activity relationship of bostrycin, Hong Chen et al. (2012) synthesized 18 bostrycin derivatives through structural modification at positions 2, 3, 6, and 7. And found that dioxylcarbonyl groups at C-2 and C-3 positions, tertiary amino groups at C-6 position and alkylthio groups at C-6 and C-7 positions of the bostrycin could enhance cytotoxicity of bostrycin derivatives.

#### Anti-Tumor Activity of Bi-Tetrahydroanthraquinones

Alterporriol L could effectively inhibit the proliferation and growth of breast cancer cell line MCF-7 (IC_50_, 13.11 μM) and MDA-MB-435 cells (IC_50_, 20.04 μM), and there was a dose-dependent manner of cell death. Moreover, alterporriol L could induce cancer cell apoptosis and necrosis through triggering the generation of oxidative stress (Huang et al., 2012).

It is interesting that we have not found any report of hydroxyphenanthrenes with anti-tumor activity. This may suggest that the tetrahydroanthraquinone skeleton with carbonyl groups at C-9 and C-10 positions is important for the anti-tumor activity of tetrahydroanthraquinones.

### Antimicrobial Activity

Many tetrahydroanthraquinones exhibit good antimicrobial activities. Altersolanols A–C and E inhibited the growth of all Gram-positive bacteria and *Pseudomonas aeruginosa* IF0 3080 with minimum inhibitory concentration (MIC) value ranging from 12.5 to 25 μg/ml. However, Altersolanol D and F, lack of hydroxy group at C-5 compared to Altersolanols A–C and E, even at concentration as high as 100 μg/ml, have not inhibited bacteria growth. This indicated that the hydroxy group at C-5 position was necessary to the antibacterial activities of tetrahydroanthraquinones (Yagi et al., 1993). Coniothranthraquinone 1 showed antibacterial activity against *Staphylococcus aureus* ATCC 25923 (SA) and *Staphylococcus aureus* SK1 (MRSA), with MIC values of 16 and 8 μg/ml, respectively (Khamthong et al., 2012; Ng et al., 2015). While trichodermaquinone showed antibacterial activity against MRSA, with a MIC value of 200 μg/ml, had no inhibition on SA (Khamthong et al., 2012). This study suggested that the hydroxyl group at C-5 position and the methyl group at C-7 position are important to the antibacterial activity of a tetrahydroanthraquinone. Deoxybostrycin and Bostrycin showed strong antimicrobial activities against *Staphylococcus aureus*, *Escherichia coli*, *Pseudomonas aeruginosa*, *Sarcina ventriculi*, *Bacillus subtilis* with an IC50 of 3.13 µg/ml, and inhibited *Candida albicans* with an IC50 of 12.5 µg/ml. Besides, Deoxybostrycin showed good anti-mycobacterial activity, it exhibited a better inhibitory effect on clinical multidrug-resistant *M. tuberculosis* (K2903531 and 0907961) than the first line anti-tuberculosis drug (Nigrosporin) (Yagi et al., 1993). Alterporriol G showed antibacterial activity only against *Streptomyces pneumonia* (Aly et al., 2008).

### Antiviral Activity

Tetrahydroaltersolanol C exhibited a significant anti-PRRSV (Porcine reproductive and respiratory syndrome virus) activity with a EC_50_ value of 12.11 µM, it inhibited the internalization and replication of PRRSV, but did not directly inactivate the virus or block its adsorption to cell surface (Zhang S.L. et al., 2016).

### Antidiabetic Activity

4-des-hydroxyl altersolanol A significantly reduced the level of blood sugar in alloxan-induced diabetic mice (Uzor et al., 2017).

## Conclusions and Perspectives

Anthraquinone compounds and their natural derivatives, especially tetrahydroanthraquinones, showed a considerably wide range of pharmacologica1 effects, and 60 tetrahydroanthraquinones have been found since altersolanol A was originally reported in 1967 (Stoessl, 1969b). Some of them exhibited considerable cytotoxicity, antimicrobial, antiviral activity, and hypoglycemic activities. In this review, anti-tumor, anti-microbial, antiviral activity, and anti-diabetic activities of tetrahydroanthraquinones are summarized in detail, and 17 active ones are involved. We try to sum up the structure and activity relationship of tetrahydroanthraquinones from previous literatures.

Anthraquinones exhibited potent antitumor effect, while they also with DNA toxicity, can be inserted into the helical structure of DNA in the form of a flat structure, affecting the transcription and DNA replication (Adhikari and Mahar, 2016). Hence, anthraquinones showed stronger toxicity than pharmacological effect. Tetrahydroanthraquinones, especially tetrahydro-9,10-anthraquinones, avoided DNA toxicity caused by anthraquinone planar structure. Tetrahydroanthraquinone forms a dimensional construct of cyclohexene after hydrogenation, and forms two or more chiral centers by substitution with OH or CH_3_. This increased the potential druggability and formed multiple targeting centers, providing more possibilities for chemical modification and structure transformation. There are few reports in the available literature that the antitumor effect of tetrahydroanthraquinone is related to DNA toxicity, except for (1S,3S)-austrocortirubin (Wang Y. et al., 2015). More studies suggested that the antitumor effect of tetrahydroanthraquinone is through targeting signaling pathways, including NF-κB, PI3K/Akt pathway (Xie et al., 2010; Zhu et al., 2012; Genov et al., 2016). Suggesting that the antitumor mechanisms of tetrahydroanthraquinones are different from that of anthraquinone. Tetrahydroanthraquinones deserve more attention and more research.

For anti-cancer activity, it seems that the p-quinone moiety of tetrahydroanthraquinone is fundamental, as reduction of one of the carbonyl groups of the quinone moiety nullified the cytotoxicity of the tetrahydroanthraquinone derivatives (Zhang N. et al., 2016). Adding short side chains to the benzoquinone increases cytotoxicity of tetrahydroanthraquinones, whose cyclohexyl ring is substituted with two hydroxyl groups with the appropriate stereochemistry, and elimination of both or even a single hydroxyl, or change of stereochemistry of the tertiary hydroxyl eliminates biological activity, and additive hydroxyl moiety at C-1 of a tetrahydroanthraquinone might be the active profile for inhibiting lung tumor cell growth. Linker at a 3-atom with a phenyl or para-chlorophenyl moiety also can enhance cytotoxicity (Phunlap et al., 2013; Teiten et al., 2013; Li and Jiang, 2018). For anti-microbial activity, from the few studies, the hydroxyl group on the C-5 might be crucial to the anti-microbial activity (Yagi et al., 1993).

The most pharmacological researches of tetrahydroanthraquinones focused on cytotoxicity and antitumor mechanisms, suggesting its potential for developing antitumor drugs. While we found some of them are not suitable for drug research. Bi-tetrahydroanthraquinones are not suitable for drug research due to large weight and large steric resistance. Tetrahydro-5,8-anthraquinones are a class of rare compounds with unstable structure, with tautomerism at the 9, 10 positions of its 5,8-dione. They are not suitable for drug development due to the difficult in structural modification and pharmacological research. Hydroxyphenanthrenes is characteristic by the C9 carbonyl group undergoes a reduction reaction to form a hydroxy substitution. However, under acidic conditions, the C9 carbonyl group undergoes dehydration reaction, and becomes keto group. therefore, special attention needs to be paid to the conditions in pharmacological and synthetic research of Hydroxyphenanthrenes.

The tetrahydroanthraquinone isolated and identified are mainly from endophytes, and some of them are isolated from marine fungi and plants. Some pharmacological activities of tetrahydroanthraquinones are reported, yet are not enough. There is still a strong possibility that some tetrahydroanthraquinones with better regulatory activities remain in the shadow or have not been fully studied. Our study will serve as a valuable guideline for further research on the structural optimization, mechanism study, and development of tetrahydroanthraquinone as novel drugs.

## Author Contributions

SF: Manuscript writing and figure preparation; WW: Literature search and manuscript editing.

## Funding

This research was funded by Natural science foundation of Guangdong Province (No.2016A030313034), Natural Science Foundation of Fujian Province (2018J01064), the Foundation of Third Institute of Oceanography SOA (2018021 and 2017001), COMRA program (DY135-B2-05 and DY135-B2-01).

## Conflict of Interest

The authors declare that the research was conducted in the absence of any commercial or financial relationships that could be construed as a potential conflict of interest.
